# Lung ultrasound reaeration score: a useful tool to predict non-invasive ventilation effectiveness

**DOI:** 10.1186/cc13445

**Published:** 2014-03-17

**Authors:** L Nobile, P Beccaria, M Zambon, L Cabrini, G Landoni, A Zangrillo

**Affiliations:** 1 Ospedale San Raffaele, Milan, Italy

## Introduction

The aim of our study is to evaluate the lung ultrasound (LUS) reaeration score (ReS) as a predictive tool for non-invasive ventilation (NIV) efficacy in general wards for acute respiratory failure (ARF) treatment. Even if ICUs are considered the safest place to perform NIV, a shortage of intensive beds has lead to NIV application outside the ICU. With appropriate patient selection, treatment-timing choice, adequate monitoring and staff training, NIV application in general wards can allow one to safely treat patients at an early stage with better cost-effectiveness [1]. Few data assessing the right tool to evaluate NIV treatment efficacy are available.

## Methods

We present preliminary data of a prospective observational ongoing study. Sixteen patients undergoing NIV treatment outside the ICU for ARF of any origin were evaluated with LUS at three times: before NIV application (T0), and at 5 minutes (T5) and 60 minutes (T60) of NIV treatment. US scan was performed in six regions for each emithorax. LUS patterns were defined as: consolidation (C); multiple coalescent B-lines (B+); multiple irregularly spaced B-lines (B) and normal aeration (A). A LUS-ReS [2] was calculated detecting changes in the US pattern when comparing T0 to T5 and T0 to T60 assessments. Outcome was defined as NIV failure in the case of tracheal intubation or death within 1 week from ARF outset, otherwise NIV success.

## Results

**Figure 1:**
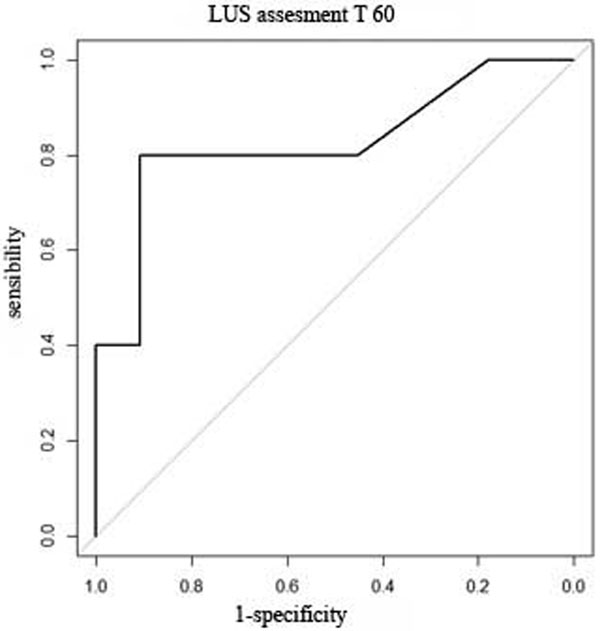


NIV treatment failed in five patients. Eleven patients have been successfully treated with NIV. Mean LUS-ReS (SD) at T0 to T5 was 0 (± 3.1) in group 0 and 2.5 (± 2.5) in group 1 (*P *= 0.15). Mean LUS-ReS (SD) at T0 to T60 was -1.2 (± 3.9) in group 0 and 4.2 (± 3.4) in group 1 (*P *= 0.03). ROC curves were obtained for the two LUS-ReS at T0 to T5 (AUC 0.72) and T0 to T60 (AUC 0.83) (Figure 1). A LUS-ReS cutoff value of 0 can predict NIV effectiveness, with a sensibility of 91% and a specificity of 80%.

## Conclusion

If confirmed, our preliminary results suggest that LUS-ReS could be a useful tool in predicting NIV effectiveness for ARF treatment. Caution has to be applied when interpreting our results considered the small amount of patients enrolled.
